# Cullin Ring Ubiquitin Ligases (CRLs) in Cancer: Responses to Ionizing Radiation (IR) Treatment

**DOI:** 10.3389/fphys.2019.01144

**Published:** 2019-10-01

**Authors:** Shahd Fouad, Owen S. Wells, Mark A. Hill, Vincenzo D’Angiolella

**Affiliations:** ^1^Medical Research Council Institute for Radiation Oncology, Department of Oncology, University of Oxford, Oxford, United Kingdom; ^2^Genome Damage and Stability Centre, School of Life Sciences, University of Sussex, Brighton, United Kingdom

**Keywords:** cullins, ionizing radiation (IR), double-strand breaks (DSBs), DNA-damage, cullin ring ligases (CRLs), E3-ligases

## Abstract

Treatment with ionizing radiation (IR) remains the cornerstone of therapy for multiple cancer types, including disseminated and aggressive diseases in the palliative setting. Radiotherapy efficacy could be improved in combination with drugs that regulate the ubiquitin-proteasome system (UPS), many of which are currently being tested in clinical trials. The UPS operates through the covalent attachment of ATP-activated ubiquitin molecules onto substrates following the transfer of ubiquitin from an E1, to an E2, and then to the substrate *via* an E3 enzyme. The specificity of ubiquitin ligation is dictated by E3 ligases, which select substrates to be ubiquitylated. Among the E3s, cullin ring ubiquitin ligases (CRLs) represent prototypical multi-subunit E3s, which use the cullin subunit as a central assembling scaffold. CRLs have crucial roles in controlling the cell cycle, hypoxia signaling, reactive oxygen species clearance and DNA repair; pivotal factors regulating the cancer and normal tissue response to IR. Here, we summarize the findings on the involvement of CRLs in the response of cancer cells to IR, and we discuss the therapeutic approaches to target the CRLs which could be exploited in the clinic.

## Introduction: The Cullin Ring Ubiquitin Ligases

Ubiquitylation is a versatile post-translational modification to control protein levels in cells and modulate cellular states to allow adaptation under stress. Indeed, this secondary modification is crucial for the cellular response to DNA damaging agents including (Ionizing Radiation) IR (Schwertman et al., 2016). The classical process of ubiquitylation is based on the activation of a ubiquitin molecule by ATP, followed by its covalent attachment to an E1 enzyme, then its transfer from the E1 enzyme to an E2 enzyme, and finally its transfer from the E2 enzyme onto selected substrates (Hershko et al., 1983; Finley et al., 2004). Selection of substrates is operated by the E3 enzymes, which bridge substrates to the E2 enzyme for ubiquitin transfer. The human genome codes for 2 E1s, ∼35 E2s and more than 700 E3 ubiquitin ligases. The organization of the system is hierarchical which allows for substrate specificity and, at the same time, diversity in substrate modification.

**FIGURE 1:**
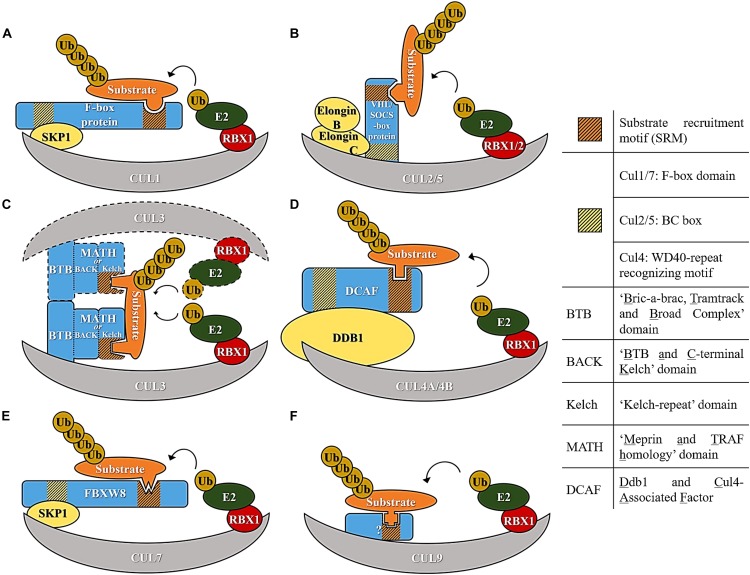
Diagram representing E3 ubiquitin ligase complexes of the cullin family of E3 ligases. **(A)** Cul1-based E3 ligase. **(B)** Cul2/5-based E3 ligase. **(C)** Cul3-based E3 ligase. **(D)** Cul4-based E3 ligase. **(E)** Cul7-based E3 ligase. **(F)** Cul9-based E3 ligase.

Cullin ring ubiquitin ligases are multi-subunit E3 ubiquitin ligases which use a specific cullin as a central scaffold to bridge an E2 enzyme to the substrate. All the cullin complexes use a cullin C-terminal portion to recruit a RING-Box protein (Rbx1 or Rbx2), required for the interaction with an E2. The N-terminal portion of the cullin binds to a different adaptor protein containing a domain that interacts with a range of variable substrate recruitment subunits, specific to that culllin class. In general, an adaptor protein, different for each cullin, links the cullin N-terminus to the aforementioned variable substrate recruitment subunit (Sarikas et al., 2011). The specific complexes formed by Cul1, Cul2, Cul3, Cul4, Cul5, Cul7, and Cul9 are highlighted in Figure 1 and are individually discussed below.

The prototypical multi-subunit E3s are Cullin1-based E3s, which assemble an SCF complex composed of Skp1, Cul1, and an F-box protein. In this particular E3 machinery, the Cul1 N-terminus binds to Skp1 (adaptor subunit), which in turn binds to a variable F-box protein (Figure 1A). F-box proteins derive their name from cyclin F in which the F-box domain, required for interaction with Skp1, was first discovered (Bai et al., 1996). After the discovery of cyclin F, about 69 F-box proteins were identified in the human genome based on their interaction with Skp1 and sequence homology within the F-box domain (Cenciarelli et al., 1999; Winston et al., 1999). F-box proteins were further sub-classified according to the protein domains they use to recruit substrates. Three F-box subfamilies exist; a subfamily that has a WD-40 domain (Fbxws), another that has a Leucine zipper (Fbxls) and finally one that has other variable domains (Fbxos) (Jin et al., 2004) (Figure 1A).

Work in the nematode *C. elegans* revealed the presence of cullin genes highly related and similar to Cul1. A search for EST in the human database identified Cul1, Cul2, Cul3, Cul4A, Cul4B, and Cul5 genes (Kipreos et al., 1996). Although the general organization of Cul2, Cul3, Cul4A, Cul4B, and Cul5 resemble the SCF assembly, structural studies have revealed substantial differences in the assembly and use of adaptors. These different features likely reflect different biochemical properties and mechanisms of action/ubiquitylation, however, studies on the kinetics of action comparing Cul1 to Cul2, Cul3, Cul4A, Cul4B, and Cul5 are lacking.

Cul2 and Cul5 are the most structurally related among the cullins and use elongins B and C as adaptors to engage a variable substrate recruitment protein. Among the most well-studied substrate recruitment proteins are the VHL tumor suppressor protein and VHL-like proteins, which use Cul2 as scaffold. Cul5 also recruits elongins B and C as adaptors but assembles with SOCS proteins to form a functional E3 (Figure 1B). The difference in specificity between Cul2 and Cul5 is related to the presence of a Cul2 and a Cul5 box, and these two distinct sequences mediate the interaction with the substrate recruitment subunits (Kamura et al., 2004).

In Cul3 complexes several BTB domain-containing proteins assemble directly with Cul3 and act as both an adaptor and a substrate recognition subunit. The BTB domain-containing proteins recognize substrates with their MATH (Meprin and TRAF homology) motif and Kelch beta-propeller repeats (Pintard et al., 2004; Genschik et al., 2013). A characteristic of these complexes is their intrinsic capacity for assembling homo-dimers through the BTB (Figure 1C). A quality control system regulating homo-heterodimerization of Kelch like proteins (Klhls) has recently been unveiled and depends on the activity of another E3 ubiquitin ligase of the F-box clade (Fbxl17) (Mena et al., 2018).

Cul4 machineries, which comprise Cul4A and Cul4B, use Ddb1 as an adapter. Ddb1 contains three WD40 propeller domains (BPA, BPB, and BPC) and assembles with a large family of DCAF (Ddb1 and Cul4 Associated Factor) proteins using a WDXR motif within the substrate recruitment factors (Figure 1D) (Jin et al., 2006).

Cul7 is similar to Cul1 in using Skp1 as an adaptor and recruiting Fbxw8 as a substrate receptor (Dias et al., 2002), but it can only assemble with Fbxw8 and not other F-box proteins (Figure 1E). The last member of the family and the most recently identified is Cul9 for which features of a cullin assembly are less clear (Figure 1F).

An attribute of the majority of the substrate recruitment subunits of the CRLs is that they recognize modified or unmodified short and unique amino acid sequences in substrates to initiate substrate engagement and ubiquitylation. These are collectively termed “degrons,” as they mark proteins for proteasomal degradation by the respective cullin machinery. Degrons are well-established but only for a small fraction of CRLs and novel insights have been recently made in deciphering the degron code at a system-wide level (Koren et al., 2018). Although the general organization of CRLs is conserved across different cullins there are substantial differences in complex assembly, which could dictate diverse modes of substrate engagement and modification. As more details of substrate engagement and ubiquitin chain specificity emerge, it will be important to compare the kinetics of action as well as the specificities of the different CRL complexes.

CRL complexes have been linked to many aspects of tumorigenesis as they participate in multiple biological processes. This review focuses specifically on the role of CRLs in the cellular response to IR covering also aspects of IR responses not related strictly to DSB repair.

## Ionizing Radiation-Induced Damage

IR can induce a wide variety of biological effects within the cells and tissues and there is strong evidence to suggest that DNA damage is a major consequence. Indeed, a typical 2 Gy X-ray fraction used in clinical radiotherapy will result in approximately 80 DSBs, 2,000 SSBs, and over 4,000 base damages (Shrieve and Loeffler, 2011). Interestingly, the number of DNA lesions produced is low in comparison to the background level of endogenous DNA damage which corresponds to the order of 50,000 DNA lesions per day as a result of Reactive Oxygen Species (ROS) and other reactive metabolites (De Bont and van Larebeke, 2004). A critical feature of all types of IR is the deposition of energy in the form of highly structured tracks of ionization and excitation events and is therefore highly heterogeneous in space and time (Hill, 2018). This results in clusters of events not only on the nanometer scale, but in some cases on the micrometer scale, which could be responsible for the effectiveness of IR in treating cancers.

Damage to DNA is produced either by direct ionization of constituent atoms or indirectly as a result of free radicals produced close by in the surrounding water. This indirect damage is dominated by the hydroxyl (OH) radical which has a lifetime of ∼4 ns and a diffusion distance of ∼6 nm within the cell (Roots and Okada, 1975). As a result of clustering, these events not only produce simple isolated lesions such as base damage, abasic sites, SSBs, DNA-protein cross-links, but importantly are also efficient at producing combinations of these, including complex SSBs, DSBs, or complex DSBs. The increase in complexity of these DSBs results in an increase in the lifetime of these lesions and a decrease in overall repairability (Eccles et al., 2011). The relative proximity of these breaks can subsequently result in a wide range of chromosome aberrations including deletions, and for many cell types is an important driver of cell death as a result of a mitotic catastrophe.

While DNA damage produced is likely to dominate the response following clinical exposures, it is also known that IR can perturb existing intra- and inter-cellular signaling which may result in a change in the background level of ROS. These changes may be temporary but can often result in a persistent change in homeostasis of this signaling, and associated changes in oxidative stress can lead to a modulation of the background rate of endogenous DNA damage induction. Almost all solid tumors contain oxygen-deficient regions and as a result oxygen, or more specifically the absence of it, can play a significant role in the radiation response of tumors. The presence of oxygen at the time of irradiation results in an enhancement in the yield and complexity of the initial damage produced over that produced under hypoxia, as a result of the oxygen chemically “fixing” the damage thereby making it permanent (Rockwell et al., 2009). In addition to modulating the initial DNA damage produced by radiation, hypoxia results in the dysregulation of oxygen homeostasis and activates pathways which alter the phenotype of the cell, adapting the cell to the stress associated with low oxygen levels.

## Roles of Cul1 in the Cellular Response to IR

### Cul1 and IR-Induced Cell Cycle Arrest

Cells entering the cell cycle duplicate their DNA in S phase and complete cell division in M phase. The presence of checkpoints allows fidelity of cell cycle transitions. For instance, when cells are exposed to IR, the DNA damage generated promotes the activation of a checkpoint response. Cell cycle progression restarts only when the DNA damage is repaired.

The progression from one cell cycle phase to the next is promoted by the periodic oscillations of cyclins that partner with CDKs to phosphorylate target substrates (Murray, 2004). The activity of CDKs is halted by phosphorylation of the CDK subunit on tyrosine 15 mediated by the tyrosine kinase Wee1 and reverted by the Cdc25A phosphatase. The presence of DNA damage induced by IR activates checkpoint kinases (Chk1 and Chk2) whose role is to prevent the activity of CDKs and arrest cell cycle progression to allow DNA repair. Chk1 performs this function by controlling the ubiquitylation and subsequent degradation of the Cdc25A phosphatase (Busino et al., 2003; Jin et al., 2003). Cdc25A activates CDKs by removing the Wee1-mediated inhibitory phosphorylation on Tyr15 of CDKs. Therefore, upon activation of Chk1, the degradation of Cdc25A prevents CDK activity which in turn restrains DNA replication and progression of the cell cycle (Bartek and Lukas, 2003).

Beta Transduction Repeat-Containing Proteins β-TrCP1/Fbxw1 and β-TrCP2/Fbxw11, jointly referred to as β-TrCP, are the two Cul1 E3 ubiquitin ligases promoting the degradation of Cdc25A (Busino et al., 2003; Jin et al., 2003). A feature of the SCF^β−TrCP^ ubiquitin ligase is that it recognizes substrates containing the degron sequence DSGXXS in which both serines need to be phosphorylated (Guardavaccaro et al., 2003). Chk1 promotes the phosphorylation of Cdc25A and its subsequent degradation but the specific kinases responsible for Cdc25A degron phosphorylation are not completely clear. A number of kinases have been proposed to phosphorylate Cdc25A in addition to Chk1. Cdc25A, unlike the majority of other β-TrCP substrates that have the consensus degron DSGXXS, has the alternate degron ^76^SSESTDSG^83^ with serines Ser76, Ser79, and Ser82 phosphorylated (Busino et al., 2003; Jin et al., 2003). CkIα was shown to phosphorylate Ser76 of Cdc25A (Honaker and Piwnica-Worms, 2010), while Nek11 was shown to phosphorylate Ser82 (Melixetian et al., 2009). Gsk3β can carry out the priming phosphorylation of Ser76 on the Cdc25A degron (Kang et al., 2008).

The choice of kinase could be influenced by the cell cycle phase, for example Nek11-phosphorylation of Cdc25A is most active in S and G2 phases and is required for the establishment of a G2/M checkpoint promoted by IR, whereas Gsk3β and Chk1 are active in G1 and S phases. The control of Cdc25A by β-TrCP following DNA damage is crucial for normal cell cycle progression and the checkpoint response. Therefore, it represents a node with multiple control points. The lack of β-TrCP and Cdc25A degradation forces cells to undergo DNA replication in the presence of DNA damage, giving rise to a subsequent mitotic catastrophe and cell death (Busino et al., 2003; Jin et al., 2003).

In addition to Cdc25A, β-TrCP regulates other crucial components of the checkpoint response: Claspin and Wee1. Wee1 counteracts the activity of Cdc25A on CDK1. It follows that since β-TrCP is simultaneously acting on Wee1 and Cdc25A, the net effect of blocking β-TrCP should have no overall impact on cell cycle progression. However, the consequences of β-TrCP knockdown are significant. The discrepancy could be explained by the different-timed action of β-TrCP on Cdc25A and Wee1 dictated by the kinases responsible for phosphorylating the respective degrons. The kinases phosphorylating Cdc25A are acting when the checkpoint is operating, whereas the kinases phosphorylating Wee1 are Polo Like Kinase 1 (Plk1) and CDK1 itself, whose activity marks mitotic entry and, thus, checkpoint resolution (Watanabe et al., 2004).

An important sensor of DNA damage functioning to sustain the checkpoint response is Claspin. Claspin is required for Chk1 activity prompted by the ATR kinase, which detects the presence of DNA damage. Claspin levels are also regulated by β-TrCP, and Plk1 kinase triggers Claspin degradation (Mailand et al., 2006; Peschiaroli et al., 2006). This β-TrCP-mediated degradation of Claspin prevents any further activation of Chk1 during recovery from genotoxic stress. The process facilitates the restart of the cell cycle once the DNA damage has been resolved. The leftover active Chk1 is targeted for degradation by the F-box protein Fbxo6. Fbxo6 regulates Chk1 degradation to terminate the checkpoint response in S phase (Zhang et al., 2005; Zhang Y.-W. et al., 2009). From the regulation of Cdc25A, Wee1, Claspin and Chk1 by β-TrCP and Fbxo6, a temporally-ordered picture of checkpoint activation and resolution emerges where the action of kinases is enforced and coordinated by E3 ubiquitin ligases to promote irreversible cell cycle phase transitions.

Interestingly, Liu and colleagues reported that Matrix Extracellular Phosphoglycoprotein/Osteoblast Factor 45 (Mepe/Of45) is a co-factor of Chk1. Upon knocking down Mepe/Of45 in transformed rat embryo fibroblasts, Chk1 levels decrease which sensitizes cells to IR and other DNA damaging agents. It is suggested that the way Mepe/Of45 achieves that is by competing with the E3 ubiquitin ligases binding to Chk1, therefore decreasing the degradation of Chk1 by the UPS (Liu et al., 2009). However, more detailed mechanistic insights are necessary to derive a clear picture of the role of Mepe/Of45.

Another Cul1 adaptor with important roles in the IR-induced checkpoint response is cyclin F (Fbxo1). It has been shown that cyclin F prevents mitotic entry after IR by enforcing a G2 checkpoint. The mechanism relies on the capacity of cyclin F to competitively inhibit B-Myb. B-Myb activates the transcription of a number of genes required for mitotic entry, therefore the inhibition of transcriptional activity of B-Myb by cyclin F enables cyclin F to control mitotic entry after IR (Klein et al., 2015). Our laboratory has shown that the levels of cyclin F are reduced by β-TrCP at mitotic entry to facilitate the transition from G2 to M phase of the cell cycle (Mavrommati et al., 2018). However, it is yet unclear whether the regulation of cyclin F by β-TrCP has a role in the checkpoint response induced by IR in G2 as well.

It is also important to note that cyclin F ubiquitylates Exo1 which is a 5′ to 3′ exonuclease with a significant role in DNA repair after IR (Elia et al., 2015). One of the major forms of DNA damage induced by IR is the formation of DSBs. DSBs could be repaired by two main pathways: (1) simply ligating the broken ends together by non-homologous end joining (NHEJ); (2) by using a second copy of DNA as a template to initiate homologous recombination (HR). HR includes a number of steps which enable the engagement of a second copy of DNA to use as a template. An important part of the process that commits the cells to repair through HR is the resection of the broken ends of DNA to facilitate strand invasion of the homologous template. Exo1 controls long-range resection at DSBs upon IR, a process that needs to be tightly regulated to avoid hyper or hypo activity of Exo1. Thus, Exo1 levels are regulated by both phosphorylation and ubiquitylation after IR (Tomimatsu et al., 2017). Cyclin F was shown to mediate ubiquitylation of Exo1 after cells were challenged with 4NQO, a radio-mimetic drug (Elia et al., 2015), however it is unclear whether cyclin F is participating in regulating Exo1 levels and activity (and therefore resection) after IR.

Exo1 is not the only substrate of cyclin F involved in maintaining genome stability; there is also RRM2. RRM2 assembles with the RRM1 subunit to form a functional RNR, an essential enzyme in the production of deoxyribonucleotides (dNTPs) which are the building blocks of DNA (Nordlund and Reichard, 2006). The activity of RNR must be regulated in accordance with the cell cycle to maintain balanced levels of dNTPs for optimal DNA replication and DNA repair (D’Angiolella et al., 2012).

Abnormal dNTP levels have a negative effect on genomic stability and are known to promote transformation as well as an increase in the frequency of spontaneous mutations (Kunz et al., 1994). Accordingly, the overexpression of RRM2 promotes the development of lung cancer in mice (Xu et al., 2008), and elevated levels of RRM2 are often linked to poor prognosis in different cancer types (Morikawa et al., 2010a, b; Jones et al., 2011). RRM2 is targeted for degradation by cyclin F after phosphorylation of RRM2 on Thr33 by cyclin A/CDK1. This phosphorylation of RRM2 by CDK1 represents a failsafe mechanism to destroy RRM2 only in G2/M when the bulk of DNA replication has been completed (D’Angiolella et al., 2012). RRM2 alterations have been shown to impact DSB repair by HR in yeast (Moss et al., 2010) and mammalian cells (Gustafsson et al., 2018), thus cyclin F could also impact IR response by regulating RRM2.

Two other F-box proteins involved in checkpoint activation in response to IR are Fbxo4 and Fbxo31. This is due to their ability to regulate cyclin D1 levels following IR. Cyclin D1 promotes entry into the cell cycle from G0, and the regulation of cyclin D levels is important after the genotoxic stress induced by IR to promote cell survival (Lukas et al., 1994; Pagano et al., 1994; Agami and Bernards, 2000). It has been shown that the phosphorylation of Thr286 of cyclin D1 promotes the degradation of cyclin D1 by Fbxo4 in a cell cycle-dependent manner both in the absence of genotoxic stress, and in response to DNA damage (Pontano et al., 2008). Cell cycle-linked degradation of cyclin D1 by Fbxo4 requires RAS/RAF as its upstream pathway, whereas the DNA damage-induced degradation of cyclin D1 requires the ATM pathway (Pontano et al., 2008). Similarly to Fbxo4, Fbxo31 targets cyclin D1 for degradation after phosphorylation of Thr286, but in this case the presence of DNA damage is central to promote its degradation. The levels of Fbxo31 increase after DNA damage, unlike the levels of Fbxo4. Evidence indeed suggests that Fbxo31-mediated degradation of cyclin D1 following DNA damage may explain the prompt implementation of G1 arrest for checkpoint control (Santra et al., 2009).

### Cul1 and p53-Mediated Checkpoint Response

Arguably, the most important regulator of cell fate upon checkpoint responses is the tumor suppressor and transcription factor p53. Upon IR, p53 is activated to promote the transcription of thousands of genes required for multiple cellular functions that range from activation of cell death and immune responses to checkpoint control and DNA repair. The major E3 ubiquitin ligase for p53 is MDM2, which targets p53 for ubiquitylation to maintain it in an inactive state (Momand et al., 1992; Oliner et al., 1993). However, given its central role, p53 is regulated by a myriad of other mechanisms. Such complex regulation is necessary, because p53 represents a central hub for cell survival, thus, multiple diverse pathways converge to regulate its function. For instance, numerous studies uncovered links between p53 and NF-κB, a family of transcription regulators with roles in regulating proliferation, apoptosis, inflammation, and the DDR (Wang et al., 2017). Interestingly, one study showed that IκB Kinase 2 (IKK2/IKKβ) phosphorylates p53 on Ser362 and Ser366, and stimulates the SCF^β−*TrCP*^-mediated degradation of p53 (Xia et al., 2009). This is required to enforce inactivation of p53 in addition to the role of MDM2, however, biological consequences of this regulation are not yet fully elucidated.

The literature is somewhat confusing with regards to the regulation of p53 as another study suggests that MDM2 can be targeted for degradation by β-TrCP. MDM2 is phosphorylated by CkIα on multiple putative β-TrCP degrons (Inuzuka et al., 2010). This mechanism of regulation of MDM2 could be relevant after repeated DNA damage insults to control the transcriptional activity of p53. It is important to note that other researchers pointed out that what seems to be the degradation of MDM2 could just be epitope-masking by phosphorylation which blocks the epitope for the most commonly used MDM2 western blotting antibodies (Cheng and Chen, 2011).

Besides β-TrCP and MDM2, several ubiquitin ligases also target p53 for degradation like the Kelch domain-containing F-box protein, Jfk (Fbxo42). Jfk was found to inhibit p53-dependent transcription, and the depletion of Jfk was found to stabilize p53 and hence sensitizes cells to IR-induced DNA damage (Sun et al., 2009).

Among the transcriptional targets of p53, p21 has the most significant function in promoting cell cycle arrest. p21 acts as a direct inhibitor of CDKs and is required to enforce checkpoint control. Fbxl1, more widely-recognized as Skp2, targets p21 and p27 (the two CDK inhibitors) for ubiquitin-mediated proteolysis (Carrano et al., 1999). Skp2 has been linked to the regulation of a p53-dependent checkpoint response in melanoma cells through the control of p27 (Hu and Aplin, 2008). It is less clear whether Skp2 has a role in controlling checkpoint resolution through p21 degradation in G2, although Cul4-mediated degradation of p21 could also play a role (the roles of Cul4 are discussed in more detail later in this review).

Some F-box proteins are direct transcriptional targets of p53 like Fbxo22 (Vrba et al., 2008), however, the function of Fbxo22 as a p53 transcriptional target is yet unclear. It has been shown that Fbxo22 targets KDM4A for degradation. KDM4A is a histone demethylase that regulates the demethylation of histone H3 at positions K9 and K36 (Tan et al., 2011). However, a different study pointed out that Fbxo22 ubiquitylates p53 by forming a complex with KDM4A. In this case Fbxo22 was shown to recognize a methylated form of p53 and to promote the degradation of methylated p53. The degradation of p53 operated by Fbxo22 is required for the induction of senescence after DNA damage induced by IR (Johmura et al., 2016). Overall, multiple F-box proteins have been shown to regulate p53, and diversity in p53 regulation could simply reflect redundancy. However, it is also plausible that the regulation of p53 by each ligase depends on the input DNA damage signal and could lead to different transcriptional outputs and consequences for cell survival.

### Cul1 and DSB Repair

Breast Cancer Type 1 Susceptibility Protein (BRCA1) is a tumor suppressor that has a significant role in HR following DNA DSBs. BRCA1, with its obligate binding partner BARD1, has been shown to act as a marker for sister chromatid availability (Nakamura et al., 2019) and to serve as a scaffold at DSB sites to recruit several proteins involved in the DNA damage repair pathway (Huen et al., 2009). BRCA1 forms a complex with BRCA2 which is facilitated by PALB2 (Sy et al., 2009; Zhang F. et al., 2009). This complex recruits the recombinase Rad51 to DSB sites and promotes the loading of Rad51 onto single-strand DNA, ultimately leading to the repair of DSBs by HR (Bhattacharyya et al., 2000; Zhang et al., 2012). The loss of BRCA2 and BRCA1 function is frequent in breast and ovarian cancers. Cancers bearing inactivating mutations in these genes are defective in the repair of DSBs by HR. It follows that forcing the use of HR by promoting the formation of unrepairable SSBs and DSBs triggers cell death. This principle is at the basis of the synthetic lethality observed between the loss of BRCA and the use of PARPi (Helleday, 2011). The striking sensitivity of tumors lacking BRCA1 and BRCA2 is exploited in the treatment of breast and ovarian cancers with PARPi.

The regulation of BRCA1 and BRCA2 is complex and far from being fully elucidated. Lu and colleagues proposed that the F-box protein Fbxo44 targets BRCA1 for its UPS-mediated degradation through the SCF^Fbxo44^ E3 ligase complex (Lu et al., 2012). However, Fbxo44 did not localize= at damaged DNA sites and PARPi sensitivity was not tested after Fbxo44 depletion.

The F-box protein Emi1, also known as Fbxo5, has been recently discovered to be involved in controlling the BRCA1-BRCA2-Rad51 axis. In cell lines with inactivating BRCA1 mutations, SCF^Emi 1^ was found to target Rad51 for its ubiquitin-mediated degradation. However, under genotoxic stress Chk1 was found to inhibit Rad51 degradation by Emi1 through phosphorylation. The phosphorylation of Rad51 increases its affinity to BRCA2 and hence results in a surplus of Rad51, which restores HR in cells with faulty BRCA1 (Marzio et al., 2018). Given its role in regulating Rad51 proteolysis in cells lacking BRCA1, Emi1 was identified as a regulator of PARPi sensitivity in BRCA1-deficient triple-negative breast cancer (Marzio et al., 2018).

The Ku70/Ku80 heterodimer (Ku) is the initiating factor of the NHEJ pathway of DSB repair. Ku recognizes the ends of DSBs and recruits other components of the DNA damage repair pathway to the site of the break. After serving its role, Ku needs to be removed from the repaired DSB site but the mechanism of its removal is not clearly understood. Studying the egg extract of *Xenopus laevis* (African clawed frog), Postow et al. (2008) demonstrated that Ku80 is degraded in response to DSBs in a ubiquitin-dependent manner, and postulated that an SCF complex is the E3 ligase responsible for this ubiquitylation. It was shown that the K48-linked ubiquitylation step, but not the proteasomal degradation step that follows it, is required for removing Ku80 from repaired DSB sites (Postow et al., 2008). Fbxl12 was found to be the F-box protein responsible for the recruitment of the Ku80-ubiquitylating SCF complex to sites of DSBs, resulting in the degradation of Ku80 (Postow and Funabiki, 2013).

In mammalian cells other E3 ubiquitin ligases, which are not members of the CRL clade of E3s, have been shown to contribute to Ku80 removal from DSB sites. RING-Finger Protein 8 (RNF8) (Feng and Chen, 2012) and RNF126 (Ishida et al., 2017) were shown to be essential for the ubiquitylation and degradation of Ku80. RNF138 also promotes the ubiquitylation but not the degradation of Ku80 (Ismail et al., 2015).

Another F-box protein involved in DNA repair through Ku is Fbxw7. Fbxw7 has been previously found responsible for the ubiquitylation and subsequent degradation of a set of oncoproteins such as cyclin E, c-Jun and c-Myc via K48 linkage, which earned Fbxw7 recognition as a tumor suppressor (Nakayama and Nakayama, 2006; Welcker and Clurman, 2008). Adding to the functions of Fbxw7, it was recently discovered to promote DNA repair through NHEJ. Following exposure to IR, the ATM kinase phosphorylates Fbxw7 at Ser26 allowing the recruitment of the F-box protein to the DSB sites. Once at the DSB sites, Fbxw7 proceeds to K63-ubiquitylate the protein Xrcc4 at Lys296, which enhances the association of Xrcc4 with Ku and in turn promotes NHEJ. Given the ability of Fbxw7 to regulate NHEJ, inactivation of Fbxw7 promotes increased sensitivity of cancer cells to IR (Zhang et al., 2016).

## Roles of Cul2 and Cul5 in the Cellular Response to IR

### Cul2: Crucial Regulator of Hypoxia Response

Hypoxia-inducible factor 1 is a transcriptional factor expressed in hypoxic tumor cells and transiently induced in tumors as a result of oxidative stress following radiation (Semenza, 1999). Hif-1 transactivates several hypoxia-responsive genes, which results in the tumor acquiring malignant properties such as apoptotic resistance, enhanced tumor growth, invasion and metastasis (Semenza, 2003). Additionally, Hif-1 activates VEGFs and PDGFs which promote resistance to radiotherapy in endothelial cells, and also promote cell proliferation and blood vessel growth around tumors (Gorski et al., 1999; Moeller et al., 2004). Hif-1 is a heterodimer that consists of α and β subunits, and oxygen is responsible for the regulation of the α subunit (Hif-1α) at a post-translational level (Huang et al., 1998). In normoxia, proline residues in the ODD domain of Hif-1α get hydroxylated by oxygen-dependent prolyl hydroxylases (Bruick and McKnight, 2001; Epstein et al., 2001). The hydroxylation of Hif-1 serves as a signal to initiate the binding of Hif-1α to VHL. VHL is a Cul2 E3 ligase that assembles with Cul2, elongins B and C, and Rbx1, resulting in the proteasomal degradation of Hif-1α subunits. Under hypoxic conditions, the prolyl hydroxylases are inhibited, hindering the recognition of Hif-1α by the VHL protein and resulting in Hif-1 accumulation and a transcriptional response to hypoxia (Maxwell et al., 1999).

**FIGURE 2:**
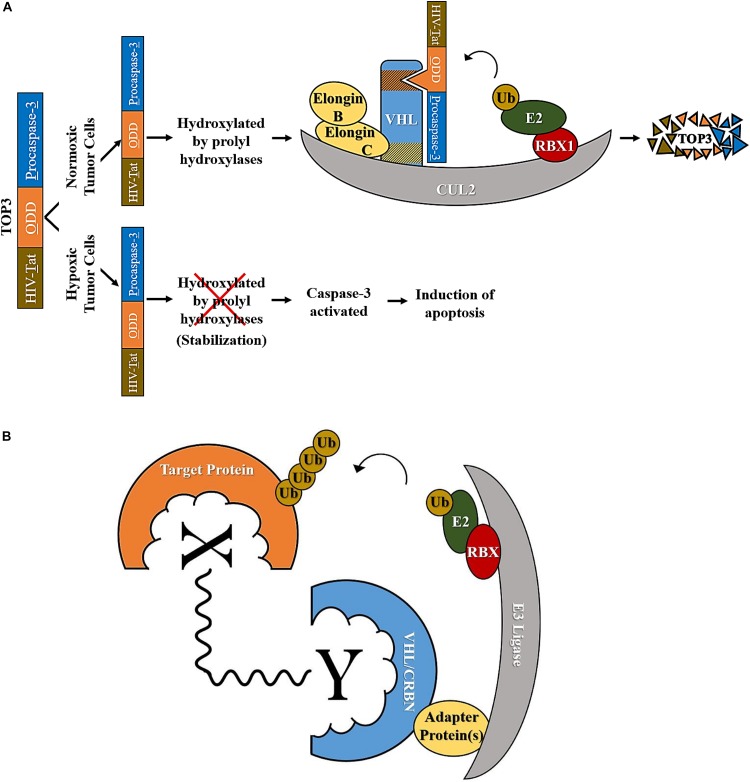
Examples of therapeutic approaches that exploit CRL complexes. **(A)** TOP3 technology; in normoxic tumor cells CRL2^VHL^ targets TOP3 for degradation while in hypoxic tumor cells TOP3 is stabilized, caspase-3 is activated, and apoptosis is induced (adapted from Harada et al., 2005). **(B)** Degradation of a target protein through bringing it in close proximity to an E3 ligase using a PROTAC (represented as X∼Y where X is the target-binding ligand, Y is the E3-binding ligand, and ∼ is the linker molecule).

Given the contribution of Hif-1α and hypoxia to malignant and radiotherapy-resistant phenotypes in cancer, researchers have been trying to eliminate Hif-1α-active cells using strategies that hijack E3 ubiquitin ligases. One strategy worth noting is the development of the fusion protein TOP3 (TAT-ODD-procaspase-3) (Figure 2A) (reproduced from Harada et al., 2002). In this three-domain protein, the first domain is constituted by the HIV-Tat protein-transduction domain, required for the efficient delivery of TOP3 to cells and tissues *in vitro* and *in vivo*. The second domain is ODD (the VHL-mediated protein destruction motif of human Hif-1α), which promotes the stabilization of the peptide in hypoxia and its degradation in normoxia. The last domain is the proenzyme form of the human caspase-3, which endows TOP3 with cytocidal activity. The caspase 3 domain is activated following accumulated-TOP3 cleavage by endogenous caspases and triggers apoptosis. TOP3 is actively eliminated by VHL in normoxic cells but is stable in hypoxic cells where it promotes cell death. Since this design enables TOP3 to target hypoxic cells for elimination while sparing normoxic cells, and IR effectively targets normoxic cells but is less effective against hypoxic cells (Brown, 1999; Vaupel, 2004), the group suggested that the combination of IR and TOP3 could be a valuable therapeutic strategy. They proceeded to test their hypothesis, and found that this combinatorial approach efficiently reduces the population of tumor cells with Hif-1α induced by both hypoxia and IR, resulting in long-term suppression of tumor growth and angiogenesis compared to treatment by either IR or TOP3 alone (Harada et al., 2002).

While additionally investigating the role of Hif-1 in the IR response, it was discovered that exposing cells to IR results in the p38 MAPK-mediated stabilization of Hif-1 under both hypoxic and normoxic conditions. It was found that upon IR, Hif-1α is stabilized by the decreased half-life of its E3 ligase subunit VHL and of its hydroxylating enzymes prolyl hydroxylases 2. Findings from this study suggest that, in combination with IR, targeting Hif-1α through its MAPK-mediated stabilization possibly has the potential to increase the efficacy of IR treatment in glioma (Kim et al., 2014). Hypoxic IR-resistant cells tend to spread, often resulting in relapse after treatment, therefore inactivation of Hif-1α could have a significant therapeutic response in glioma.

### Roles of SOCS Boxes in Signaling and as Viral Adaptors

In addition to VHL, Cul2 or Cul5 can assemble with SOCS-box and ‘ankyrin repeat and SOCS box (ASB)’ proteins to form E3 ubiquitin ligase complexes that target proteins for proteasomal degradation. In complex with Cul2, elongin BC subcomplex, and Rbx1, SOCS1 accelerates the UPS-mediated degradation of Jak2 (Kamizono et al., 2001), a STAT3-activating tyrosine kinase which when inhibited using a small-molecule inhibitor sensitizes lung cancer cells to radiotherapy (Sun et al., 2011). Furthermore, SOCS1 has been shown to directly regulate the p53 response and apoptosis in T cells after IR by modulating STAT signaling (Calabrese et al., 2009). SOCS1 was also found to act as a substrate-binding motif targeting the HPV oncoprotein E7, inducing its ubiquitylation and subsequent degradation (Kamio et al., 2004). HPV is implicated in the formation of a subgroup of head and neck cancers that display high sensitivity to radiotherapy both *in vitro* and *in vivo* (Kimple et al., 2013; Dok et al., 2014; Sorensen et al., 2014). Therefore, the inhibition of SOCS1 could further enhance E7 levels to promote radiosensitivity.

Some SOCS-box-containing viral proteins such as the HIV-1 Vif have the capacity to hijack Cul5 E3 ubiquitin ligase complexes through which they acquire the ability to degrade host proteins (Yu et al., 2003). Acting as the substrate-specificity subunit of a CRL5 complex, the HIV-1 Vif protein targets the cellular intrinsic restriction factor APOBEC3G for proteasomal degradation (Yu et al., 2004). It has been reported that the inhibition of APOBEC3G activity using a Vif-derived peptide (Vif25-39) hinders DNA DSB repair in lymphoma cells, and hence sensitizes the cells to IR. The results from this study suggest that the peptide-mediated inhibition of APOBEC3G may be a potential radiosensitizing strategy (Prabhu et al., 2016), however, the mechanisms through which APOBEC3G affects DNA repair are not fully elucidated.

Another elongin BC-box protein is Wsb1. Kim et al. (2015) discovered that Wsb1 binds VHL and mediates its ubiquitylation and its subsequent proteasomal degradation. Hence, Wsb1 stabilizes Hif-1α by promoting the degradation of VHL under both hypoxic and normoxic conditions (Kim et al., 2015). Given that Hif-1 promotes resistance to radiotherapy, it is plausible that Wsb1 negatively influences the cellular response to IR by regulating Hif-1α, however, the role of Wsb1 in radiation sensitivity remains to be investigated.

Interestingly, it was found that in glioblastoma (GBM) cell lines, SOCS1 and SOCS3 are regulated reciprocally and SOCS3 is highly expressed in GBM, while SOCS1 is not. Expressing SOCS1 promotes radiosensitivity, while SOCS3 expression activates prosurvival signals in cancer cells through STAT-signaling, specifically STAT3 (Zhou et al., 2007; Sitko et al., 2008). Baek et al. (2016) evaluated the effect of the drug Resveratrol, a known suppressor of STAT3 signaling, on apoptosis induction and radiosensitization in SCCHN. They found that Resveratrol induces the expression of the SOCS1 protein and also its mRNA, which blocks the STAT3 signaling pathway, induces apoptosis and even enhances the rate of apoptosis when combined with IR (Baek et al., 2016). However, the functions of SOCS1 and SOCS3 in regulating STAT3 signaling in terms of their capacity to form Cul5 complexes are yet to be unraveled.

## Roles of Cul3 in the Cellular Response to IR: DNA Repair and ROS Regulation

Bric-a-brac/tramtrack/broad complex -domain proteins serve as substrate-recruitment motifs of E3 ubiquitin ligase complexes and function as bridges to connect Cul3 and substrates. They have crucial roles in regulating two aspects of the IR response: DNA repair and ROS quenching.

An important aspect of DNA repair at DSBs is the choice between HR and NHEJ. While NHEJ is considered a fast and error prone repair mechanism, HR is considered error-free. However, while NHEJ can occur throughout the cell cycle, HR can only be carried out if a homologous template is present and thus is restricted to S and G2 phases of the cell cycle. Multiple mechanisms controlled by the ubiquitin system exist to control repair choice at specific phases of the cell cycle. For instance, Klhl15, a Cul3 adaptor, was found to accelerate the UPS-mediated degradation of the DNA endonuclease CtIP (Ferretti et al., 2016). CtIP together with the Mre11-Rad50-Nbs1 (MRN) complex control the choice of DNA DSB repair pathway by initiating DNA end-resection, which commits cells to HR by inhibiting NHEJ as processed DSB ends can no longer be targeted by the NHEJ pathway (Sartori et al., 2007; Chapman et al., 2012; Ceccaldi et al., 2016). Since IR induces DSBs, and CtIP is an essential factor of the DDR, the regulation of CtIP turnover by CRL3-Klhl15 is required to fine-tune the balance between HR and NHEJ in the cellular response to IR. Depletion of Klhl15 sensitizes cells to IR by disrupting the balance between these two repair pathways (Ferretti et al., 2016).

Another BTB protein that is crucial for responses to IR is Kelch-like ECH-Associated Protein 1 (Keap1). Keap1 has been directly implicated in the control of DSB repair; Keap1 ubiquitylates the BRCA1-interacting site of PALB2, preventing the interaction of PALB2 and BRCA1 and hence prohibits HR. The ubiquitylation by Keap1 does not lead to PALB2 proteolysis but restricts the interaction with BRCA1. The ubiquitylation of PALB2 is counteracted by USP11 during G2. This mechanism prevents the execution of HR in G1 (Orthwein et al., 2015).

The most well-studied function of CRL3-Keap1 is the regulation of the transcription factor Nf-E2-Related Factor 2 (Nrf2), a master mediator of the cellular defenses against ROS (Cullinan et al., 2004; Kobayashi et al., 2004; Zhang et al., 2004). Nrf2 binds to the ARE found within the promoters of several genes encoding proteins that have crucial roles in the anti-oxidation responses (Kensler et al., 2007). Under oxidative stress, several cysteine residues in Keap1 are covalently modified by thiol intermediates activated by the ROS radicals. The chemical modifications in Keap1 prevent the binding to Nrf2, which, as a consequence, is stabilized and can transcribe the genes required for the anti-oxidation response.

As oxidative stress is a key player in carcinogenesis, the Keap1-Nrf2 pathway serves a chemopreventive role and protects healthy cells from carcinogenesis. However, cancer cells have the ability to hijack this pathway to acquire survival advantages under high ROS, which are common events in cancer tissues (Chen and Chen, 2016). It was indeed found that Nrf2 overexpression in cancer cells decreases their sensitivity to IR and chemotherapy, while Nrf2 knockdown sensitizes the cells to such treatments (Shibata et al., 2008a; Wang et al., 2008; Solis et al., 2010; Zhang et al., 2010). Nrf2 is aberrantly overexpressed in many cancers, and one reason for this is the presence of mutations in Keap1, the ligase targeting Nrf2 (Shibata et al., 2008b; Yoo et al., 2012). Loss-of-function mutations in Keap1 were found in human lung carcinoma, NSCLC, gallbladder, ovarian, and liver cancers (Singh et al., 2006; Ohta et al., 2008; Shibata et al., 2008a; Konstantinopoulos et al., 2011; Yoo et al., 2012), whereas gain-of-function mutations in Nrf2 were found in lung, head and neck, and esophageal carcinoma (Shibata et al., 2008a, 2011).

Mutations in Nrf2 prevent binding to Keap1, leading to an increase in protein stability and constitutively high levels of Nrf2 (Tong et al., 2006). The mutational status of Keap1 and of Nrf2 have been directly connected to the success of localized radiotherapy in lung cancer (Jeong et al., 2017), underscoring the crucial role of this E3 ubiquitin ligase-substrate axis in the cellular response to IR.

Another protein worth noting is the chromatin-modifying enzyme Tip60; it is essential for DNA repair and apoptosis after IR (Ikura et al., 2000), its inhibition abolishes IR-induced G1 cell-cycle arrest (Berns et al., 2004), and it was also found to activate ATM following genotoxic stress (Sun et al., 2005). The stability and activity of Tip60 is reportedly tightly regulated by the combined action of Activating Transcription Factor-2 (ATF2) and CRL3, however, since ATF2 lacks a BTB domain, it is speculated that an unidentified BTB domain-containing protein is part of this CRL3 complex (Bhoumik et al., 2008).

## Roles of Cul4A and Cul4B in the Cellular Response to IR: Controlling DNA Repair and DNA Replication

Cul4 and Ddb1, the substrate adapter for CRL4 complexes (Bondar et al., 2006; Sugasawa, 2009), are conserved from yeast to humans (Holmberg et al., 2005; Bondar et al., 2006; Kim and Kipreos, 2007). In lower organisms, the Cul4 protein is encoded by a single gene, but in mammals Cul4A and Cul4B proteins are encoded by two highly homologous genes (Iovine et al., 2011). Cul4A and Cul4B are 82% identical, and the difference between the two proteins is highlighted at their N-terminals; Cul4B is 149 amino acids longer than Cul4A as it has an N-terminal extension that contains an NLS. The NLS in Cul4B is believed to mediate the nuclear localization of Cul4B whereas Cul4A is mostly located in the cytoplasm (Zou et al., 2009; Hannah and Zhou, 2015).

Cul4A is reportedly aberrantly expressed in a plethora of cancers including breast cancer, squamous cell carcinoma, pleural mesothelioma (Shinomiya et al., 1999; Yasui et al., 2002; Melchor et al., 2009; Hung et al., 2011), and lung cancer (Wang et al., 2014), and its overexpression contributes to tumor progression, metastasis and poor prognosis (Wang et al., 2014). Compared to Cul4A, findings linking Cul4B to cancer are less common, however it was still found to be overexpressed in colon cancer (Jiang et al., 2013), cervical cancer, lung cancer, esophageal cancer, and breast cancer (Hu et al., 2012), with its overexpression closely related to tumor stage, invasion and metastasis.

Again as a *leitmotiv* of all E3 ubiquitin ligases, it was reported that Cul4A associates with MDM2 to degrade p53 (Nag et al., 2004), however, dissecting the role of Cul4 from all its adaptors is quite difficult and the emerging picture is that the observed activation of p53 is likely indirect.

One role of Cul4 complexes is to recognize UV-induced pyrimidine photodimers in DNA. In this case, the Ddb1 is bound to Ddb2 and directly recognizes DNA lesions. The ubiquitylation events mediated by Ddb1-Ddb2 following recognition subsequently help recruit the required proteins for the repair of the DNA lesion. A detailed explanation of the role of Cul4 complexes in UV-irradiation is outside the scope of this review and there are excellent reviews on the topic (Scrima et al., 2011). However, it is important to mention other important Cul4 adaptors that regulate genome stability, in particular Cdt2. Cdt2 is a Cul4 substrate-recognition subunit that targets p21 (Abbas et al., 2008), p27 (Li et al., 2006), Cdt1 (Jin et al., 2006) and the histone methyl-transferase Set8 (Abbas et al., 2010) for degradation. The mode of substrate engagement that Cdt2 uses is unique: Cdt2 couples substrate degradation to DNA replication by interacting with a modified PCNA Interacting Peptide (PIP) box in the substrate.

Proliferating cell nuclear antigen is an essential DNA clamp which travels with the DNA replication fork and acts as a scaffold for the recruitment of proteins required for DNA replication completion and fidelity. Proteins that interact with PCNA have in common a PIP-box motif consisting of the following amino-acid residues: Qxxψxxϑϑ. Substrates of Cdt2 contain an equivalent PIP box, but with modifications that increase the affinity for PCNA and favor a concomitant interaction with Cdt2. Thus, the recognition of Cdt2 substrates is mediated through the PIP degron: QxxψTDϑϑxxxK/R (Havens and Walter, 2009; Michishita et al., 2011). This strategy is important to control degradation of substrates present on the chromatin and traveling with DNA replication. Indeed, Cdt2 targets chromatin-bound p21 for degradation (Abbas et al., 2008; Kim et al., 2008) whilst the soluble p21 is targeted for degradation by Skp2 (Fbxl1) (Carrano et al., 1999).

Cdt2 also targets Cdt1 for degradation during S phase. Cdt1 is an important component of the Pre-RC, which defines DNA replication origins in G1. The Cdt2-dependant degradation of Cdt1 in S phase is one of several redundant mechanisms that prevent the re-initiation of DNA replication from regions of the genome which have already been replicated (Ralph et al., 2006). Interestingly, it was previously reported that Cdt1 is degraded by a CRL4 complex upon IR. This may contribute to the inhibition of replication in response to IR, although the majority of DNA replication inhibition is ascribed to checkpoint activation (Higa et al., 2003). The turnover of Cdt2 itself is also UPS-dependent and is mediated by the E3 ligase SCF^Fbxo11^. This hints at an interesting crosstalk between CRL1 and CRL4 ligases (Abbas et al., 2013; Rossi et al., 2013).

Literature linking CRL4 complexes to IR is limited, but evidence from lower eukaryotes suggests that Cul4 based machineries could play a significant role in processing DSBs (Moss et al., 2010). More recently, Zeng et al. (2016) identified WDR70, a conserved DCAF that is recruited to DNA DSBs. WDR70 is part of a Cul4-Ddb1 ubiquitin ligase complex and promotes HR by stimulating long range resection(Zeng et al., 2016). Interestingly, knockdown of WDR70 sensitizes HEK293T cells to IR and is comparable to the survival observed upon Ddb1 depletion.

Whilst the hijacking of cullin machinery has been previously discussed, about 50% of human hepatocellular carcinomas are associated with chronic hepatitis B infection, and risk of carcinogenesis increases with viral load (El-Serag, 2011). The hepatitis B virus encodes an oncoprotein, Hepatitis B x protein (HBx) that can hijack the Cul4-Ddb1 machinery by displacing WDR70 (and likely other DCAFs) to form viral CRL4^HBx^. Disassembly of the CRL4^WDR70^ complex by HBx compromises DNA end resection and results in HR deficiency (Ren et al., 2019). Understanding the target of the CRL4^*WDR70*^ machinery may provide insights into new targets for sensitizing cancer cells to IR.

## Targeting CRL Complexes for Cancer Therapy

Given the central role that CRLs have in regulating cancer pathogenesis and therapy responses, inhibitors of CRLs with limited specificity have been developed (Jang et al., 2018). MLN4924 (pevonedistat), a small-molecule inhibitor that selectively inhibits NEDD-8, was shown to inactivate CRL complexes and suppress tumor growth both *in vitro* and *in vivo* (Soucy et al., 2009). TAS4464 is another NEDDylation inhibitor, and most clinical trials involving either MLN4924 or TAS4464 are still restricted to phase I and II trials (Jang et al., 2018). The mechanism of action of MLN4924 is not selective as MLN4924 inhibits all the NEDDylation reactions in the cells. NEDDylation is essential for ubiquitylation operated by CRLs and thus NEDDylation inhibitors are effectively blocking the action of more than 400 enzymes in the cells. Despite the limited specificity, MLN4924 was shown to sensitize cancer cells to several chemotherapeutic drugs, so several of the ongoing trials are testing this small-molecule inhibitor in combination with DNA-damaging drugs such as carboplatin (Jang et al., 2018). MLN4924 was shown to act as a radiosensitizer in a mouse xenograft model of human pancreatic cancer (Wei et al., 2012), and in the human colorectal cancer cell lines HT-29 and HCT-116 (Wan et al., 2016). It was also found that MLN4924 radiosensitizes SCCHN in culture, and enhances IR-induced suppression of SCCHN xenografts in mice (Vanderdys et al., 2018).

It is possible to specifically inhibit individual CRLs or CRL-interacting proteins such as CRL substrates or CRL substrate-recruitment units, as opposed to global CRL- or proteasomal-inhibition, and this has in fact been attempted in a number of studies. For example, targeting CRL substrates that enhance the resistance of cells to radiotherapy, or targeting CRLs whose substrates sensitize cells to IR could potentially prove as an effective strategy to radiosensitize cancer cells. Substrates and substrate-recruitment members of CRLs that are involved in the cellular response to IR are summarized below in Table 1. An alternative emerging possibility to exploit CRL roles in cancer therapy is to use PROTACs (Figure 2B). PROTACs are heterobifunctional molecules made up of two different ligands and a linker; one of the two ligands binds to the target protein, and the other binds to an E3 ubiquitin ligase (An and Fu, 2018). Upon the formation of the E3 ligase-PROTAC-protein complex, E2 enzymes ubiquitylate the target protein marking it for proteasomal degradation (An and Fu, 2018). Thereby, a PROTAC molecule increases the proximity between the target protein and an E3 ligase, and acts catalytically to induce the proteolysis of this substrate.

**TABLE 1:** Summary of the subunits of E3 ligase complexes of the cullin family that are involved in the IR response.

**Cullin family member**	**CRL substrate- recruitment subunit**	**Substrate(s)**	**References**
Cul1	β-TrCP (Fbxw1/11)	Cdc25A	Busino et al., 2003
		Claspin	Mailand et al., 2006; Peschiaroli et al., 2006
		p53	Isoda et al., 2009
		Wee1	Watanabe et al., 2004
	Cyclin F (Fbxo1)	Exo1	Elia et al., 2015
		RRM2	D’Angiolella et al., 2012
	Emi1 (Fbxo5)	Rad51	Marzio et al., 2018
	Fbxl12	Ku80	Postow and Funabiki, 2013
	Fbxo4 and Fbxo31	Cyclin D1	Lin et al., 2006; Santra et al., 2009
	Fbxo6	Chk1	Zhang F. et al., 2009
	Fbxo11	Cdt2	Abbas et al., 2013
	Fbxo22	p53	Vrba et al., 2008
	Fbxo44	BRCA1	Lu et al., 2012
	Fbxw7	Xrcc4	Zhang et al., 2016
	Jfk (Fbxo42)	p53	Uchida et al., 2009
	Mepe/Of45 (co-factor of Chk1)		Liu et al., 2009
	Skp2 (Fbxl1)	p21 and p27	Carrano et al., 1999
Cul2	SOCS1	HPV-E7	Kamio et al., 2004
		Jak2	Kamizono et al., 2001
	SOCS1, SOCS3 in GBM		Zhou et al., 2007
	VHL	Hif-1α	Maxwell et al., 1999
Cul3	Keap1	Nrf2	Zhang et al., 2004
		PALB2	Orthwein et al., 2015
	Klhl15	CtIP	Ferretti et al., 2016
	Klhl20	PML (TRIM19)	Yuan et al., 2011
	Spop		Zhang et al., 2014
		Tip60	Bhoumik et al., 2008
Cul4	Ddb1-Cdt2	Cdt1	Higa et al., 2003
		p21	Stuart and Wang, 2009
	Ddb1-WDR70		Zeng et al., 2016
Cul5	HIV-1 Vif	APOBEC3G	Yu et al., 2003
	Wsb1	VHL	Kim et al., 2015

Proteolysis-targeting chimeras were designed to overcome several of the limitations that face their predecessors, small-molecule inhibitors. Small-molecule inhibitors are the most common targeted treatment method for intracellular proteins, however, they have several shortcomings. One example of such shortcomings is that small-molecule inhibitors are mainly engineered to target proteins with active sites (ex., enzymes or receptors), whereas the majority of the proteome lacks active sites and is therefore not targetable using this method (Toure and Crews, 2016). Target protein-binding ligands in PROTACs overcome this through binding to crevices on the surface of target proteins as opposed to active sites, making a much wider range of proteins druggable (An and Fu, 2018).

Through this design, and due to the nature of PROTAC-substrate binding being transient and reversible, PROTACs are also less prone than small-molecule inhibitors to drug resistance arising from mutations at the active site of the target protein (An and Fu, 2018).

Additionally, in contrast to the relatively high concentration of small-molecule inhibitors required to reach therapeutic capacity which often leads to off-target effects, PROTACs are required at much lower concentrations for two reasons. The first is that PROTACs act sub-stoichiometrically so they are involved in multiple rounds of protein degradation as opposed to small-molecule inhibitors that act in a 1:1 ratio to the substrate (Bondeson et al., 2015; Lu et al., 2015; Olson et al., 2018). And the second reason is that the hook effect applies to PROTACs, which entails that at high PROTAC concentrations the protein degradation efficiency decreases due to the direct inhibition of the E3 target (An and Fu, 2018).

Another advantage of PROTACs over small-molecule inhibitors stems from the fact that PROTACs exploit the intracellular UPS to degrade target proteins so the whole protein is lysed, as opposed to only disrupting the activity of a single domain in a protein by small-molecule inhibitors, which leaves the other domains in a multi-domain protein fully functional.

The concept of PROTACs was first introduced in 2001 (Sakamoto et al., 2001), and the first cell-permeable PROTAC was designed a few years later in 2004 (Schneekloth et al., 2004). At their infancy, PROTACs were peptide-based but that soon changed due to the poor permeability as well as low potency of the high-molecular weight peptide ligands, and the first small-molecule PROTAC was reported in 2008 (Schneekloth et al., 2008; Lai and Crews, 2017). It was reported that immunomodulatory drugs thalidomide, lenalidomide, and pomalidomide have the ability to hijack the ubiquitin ligase CRL4^CRBN^ so small-molecule PROTACs with thalidomide (and derivatives)-based CRBN binding ligands were developed (An and Fu, 2018). Since 2015, the number of small-molecule PROTACs reported is more than 30, most of them utilizing VHL and cereblon (CRBN, a substrate recognition subunit of CRL4) E3 ligases (An and Fu, 2018). Currently, several pharmaceutical companies have programs for developing PROTACs, such companies include Arvinas, C4 Therapeutics, Kymera Therapeutics, and Captor Therapeutics (An and Fu, 2018). A large expansion of small molecules targeting E3 ligases is predicted given the significant investment from both pharma companies and academia.

## Conclusion and Future Research Directions

Given the specific roles of E3 ligase adaptors in regulating multiple aspects of the cellular responses to IR, it is possible to envision that alteration of the CRLs could be exploited to improve radiotherapy response in cancer cells. For instance, the development of PROTACs targeting CRL adaptors in the DNA damage could lead to drugs that are only activated upon IR or under a specific stimulus like hypoxia. In the future it will be important to dissect the roles of the diverse CRLs at a system-wide level. It is important to emphasize the fact that studies on some subfamilies of CRLs (like CRL4 and CRL3) and DNA damage are quite limited and require further investigations that take into consideration the role of the adaptor, the alteration in selected cancer types and the impact in therapy response with radiotherapy and other DNA damaging agents.

In the search for new approaches to improve patient response to radiotherapy, all possible ways IR affects the cell need to be considered. The effect of IR is not limited to DNA damage by DSBs and SSBs, it also affects the immune system. It was recently noted that IR treatment could facilitate the immune responses by activating a cGAS/STING pathway to initiate immune signaling (Harding et al., 2017). This observation is likely at the basis of a well-known phenomena in the clinic called the abscopal effect. The abscopal effect, first defined in 1953, is the ability of localized radiotherapy to induce off-target anti-tumor effects leading to tumor regression at non-irradiated metastatic sites (Mole, 1953). Following this discovery, researchers began looking into the mechanisms guiding such effect and only 50 years later were able to make the breakthrough proposition that it was immune-system mediated (Demaria et al., 2004). With a plethora of recent studies reporting successful oncological applications of immune checkpoint inhibitors in clinical trials, researchers are becoming increasingly interested in the effects of combining immunotherapy with localized radiotherapy to best exploit the abscopal effect (Ng and Dai, 2016; Liu et al., 2018). The cullin family of E3 ligases is involved in several pathways of immune responses (Kawaida et al., 2005; Bibeau-Poirier et al., 2006; Kuiken et al., 2012; Mathew et al., 2012; Saucedo-Cuevas et al., 2014), therefore they could also be exploited to modulate the abscopal effect and/or cGAS/STING activation in combination with radiotherapy. A recent example of the involvement of E3s in immune checkpoint responses is represented by the E3 ligase Spop. In its E3 ligase complex with Cul3, Spop targets the ligand of the immune checkpoint Pd-L1 for its ubiquitin-mediated degradation (Zhang et al., 2018). Based on this mechanism, inhibiting Spop was found to result in an abundance of Pd-L1, and in combination with anti-Pd-1 immunotherapy was found to improve tumor regression and overall survival rates in murine models (Zhang et al., 2018).

In the future, CRL adaptors could be targeted with PROTACs to enhance the response of tumors to radiotherapy and elicit a systemic immune response. Given the large expansion of PROTAC-like-molecules in the last years, it is conceivable that further studies on CRLs might improve patient survival by changing the clinical approaches to cancer treatment with radiotherapy.

## Author Contributions

SF worked on the main body of the review and collected the relevant literature. OW critically analyzed and reviewed the manuscript, and contributed to the description of the roles of Cul4 in IR responses. MH, as a radiation biophysics expert, wrote the paragraph on the biological effects of ionizing radiation. VD’A organized the literature, provided insightful comments, and revised the work on cullins.

## Conflict of Interest Statement

The authors declare that the research was conducted in the absence of any commercial or financial relationships that could be construed as a potential conflict of interest.

## Abbreviations

APOBEC3G: apolipoprotein B mRNA editing enzyme catalytic subunit 3G
ARE: antioxidant response element
ATF: activating transcription factor
ATM: ataxia telangiectasia-mutated
ATR: ataxia telangiectasia and Rad3-related
BACK: BTB and C-terminal kelch
BRCA: breast cancer susceptibility gene/protein
BTB: bric-a-brac/tramtrack/broad complex
β-TrCP: beta transduction repeat-containing protein
Cdc25: cell division cycle 25
Cdt: chromatin licensing and DNA replication factor
CDK: cyclin-dependent kinase
Chk: checkpoint kinase
CkI α: casein kinase I α
CRBN: cereblon
CRL: cullin-RING ligase
Cul: cullin
DCAF: Ddb1 and Cul4-associated factor
Ddb1: UV-damaged DNA-binding protein 1
DDR: DNA damage response
dNTPs: deoxyribonucleotides
DSB: double-strand break
Emi1: early mitotic inhibitor 1
EST: expressed sequence tag
Exo1: exonuclease 1
FDA: US Food and Drug Administration
Gsk3 β: glycogen synthase kinase 3 Beta
Hif: hypoxia-inducible factor
HIV: human immunodeficiency virus
HPV: human papilloma virus
HR: homologous recombination
IR: ionizing radiation
Jak2: Janus kinase 2
Jfk: just one F-box and kelch domain-containing protein
Kdm4A: lysine demethylase 4A
Keap1: kelch-like ECH-associated protein
Klhl: kelch-like protein
Ku: Ku70/Ku80 heterodimer
MAPK: mitogen-activated protein kinase
MATH: meprin and TRAF homology
MDM2: mouse double minute 2, human homolog of
P53: binding protein matrix extracellular
Mepe/Of: phosophoglycoprotein/osteoblast factor
MRN: Mre11-Rad50-Nbs1 complex
Nek11: NIMA (never in mitosis gene A)- related kinase 11
NF- κ B: nuclear factor kappa-light-chain-enhancer of activated B cells
NHEJ: non-homologous end joining
NLS: nuclear localization signal
Nrf2: Nf-E2-related factor 2
ODD: oxygen-dependent degradation
PALB2: partner and localizer of BRCA2
PARP: poly-ADP-ribose polymerase
PCNA: proliferating cell nuclear antigen
Pd-1: programed cell death protein 1
PDGF: platelet-derived growth factor
Pd-L1: programed cell death 1 ligand 1
Plk1: polo like kinase 1
PML: promyelocytic leukemia
PROTAC: proteolysis-targeting chimera
Rbx: RING-box protein
Rnf: RING-finger protein
RNR: ribonucleotide reductase
ROS: reactive oxygen species
RRM: ribonucleotide reductase family member
SCCHN: squamous cell carcinoma of the head and neck
SCF: Skp1/Cul1/F-box protein
Skp1: S-phase kinase-associated protein 1
SOCS: suppressor of cytokine signaling
Spop: speckle-type poxvirus and zinc-finger protein
SRM: substrate recruitment motif
SSB: single-strand break
STAT: signal transducer and activator of transcription
Tip60: 60 kDa tat-interactive protein
TOP3: TAT-ODD-procaspase-3
UPS: ubiquitin-proteasome system
Usp11: ubiquitin specific peptidase 11
VEGF: vascular endothelial growth factor
VHL: von-Hippel-Lindau
Vif: viral infectivity factor
Wsb1: WD repeat and SOCS box-containing protein 1
Xrcc4: X-ray repair cross complementing 4.
